# PEGylated ATP-Independent Luciferins for Noninvasive High-Sensitivity High-Speed Bioluminescence Imaging

**DOI:** 10.1021/acschembio.4c00601

**Published:** 2024-12-23

**Authors:** Xiaodong Tian, Yiyu Zhang, Hui-Wang Ai

**Affiliations:** † Department of Molecular Physiology and Biological Physics, University of Virginia School of Medicine, Charlottesville, Virginia 22908, United States; ‡ Center for Membrane and Cell Physiology, University of Virginia School of Medicine, Charlottesville, Virginia 22908, United States; § The UVA Comprehensive Cancer Center, University of Virginia, Charlottesville, Virginia 22908, United States

## Abstract

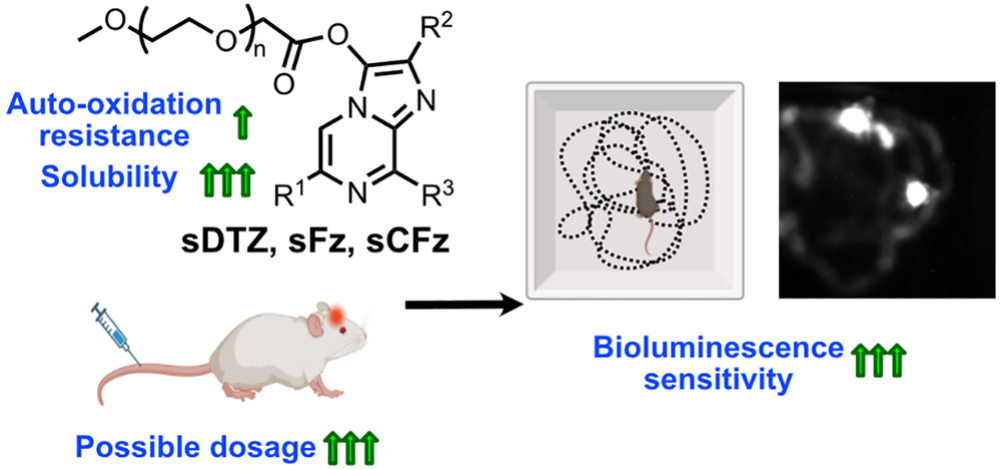

Bioluminescence imaging (BLI) is a powerful, noninvasive imaging method for animal studies. NanoLuc luciferase and its derivatives are attractive bioluminescent reporters recognized for their efficient photon production and ATP independence. However, utilizing them for animal imaging poses notable challenges. Low substrate solubility has been a prominent problem, limiting *in vivo* brightness, while the susceptibility of luciferins to auto-oxidation by molecular oxygen in air increases handling complexity and poses an obstacle to obtaining consistent results. To address these issues, we developed a range of caged PEGylated luciferins with increased auto-oxidation resistance and water solubility of up to 25 mM, resulting in substantial *in vivo* bioluminescence increases in mouse models. This advancement has created the brightest and most sensitive luciferase-luciferin combination, enabling high-speed video-rate imaging of freely moving mice with brain-expressed luciferase. These innovative substrates offer new possibilities for investigating a wide range of biological processes and are poised to become invaluable resources for chemical, biological, and biomedical fields.

## Introduction

Bioluminescence imaging (BLI), which utilizes luciferase-catalyzed oxidation of luciferins for photon production, is a powerful, noninvasive method for monitoring biological processes in animal models.^1,2^ Compared to fluorescence imaging, BLI avoids issues such as photobleaching and phototoxicity and enables sensitive signal monitoring in deep tissues with high signal-to-background ratios.^3^ Compared to alternative *in vivo* imaging modalities such as magnetic resonance imaging (MRI) and positron emission tomography (PET), BLI offers superior spatiotemporal resolution, cost-effectiveness, convenience, and the absence of radioactive contrast agents.^4,5^ When combined with bioluminescent indicators, BLI can track specific bioactivities, making it a popular imaging technique for both basic and preclinical research.^6−10^

Firefly luciferase (FLuc), which utilizes D-luciferin as its substrate, is a widely adopted bioluminescent reporter due to its long-wavelength light emission and the substrate’s chemical stability and water solubility. Research has developed FLuc and D-luciferin derivatives offering enhanced properties such as higher brightness, more red-shifted emission, and orthogonal reactivity.^1,11−14^ Of particular note is the Akaluc luciferase coupled with the AkaLumine luciferin (also known as TokeOni), recognized as a leading benchmark for deep-tissue sensitivity.^15^ However, these luciferases and their derived bioluminescent indicators inherently depend on ATP, a crucial energy source and signaling molecule, for their operation.^7,16^

Conversely, the oxidation of coelenterazine (CTZ, Figure S1A) luciferin by marine luciferases is ATP-independent. In 2012, Promega introduced NanoLuc, a marine luciferase mutant, known for its high photon production with the synthetic luciferin named furimazine (Fz, Figure S1A), small molecular size, remarkable enzyme stability, and flexibility for split and domain insertion.^17,18^ Despite its popularity, NanoLuc faces challenges for *in vivo* BLI, such as limited tissue penetration of the emitted blue photons, low substrate stability and solubility, and inadequate luciferin entry to the brain.^16,19,20^ Recent studies partially addressed these concerns by developing additional CTZ analogs and NanoLuc mutants,^21−23^ or by fusing luciferases to long-wavelength-emitting fluorescent proteins (FPs) for redder emission.^21,24−26^ Furthermore, NanoLuc and its derived luciferases have been effectively transformed into bioluminescent indicators, enabling successful imaging of Ca^2+^ and K^+^ dynamics in live mice.^26−28^ Despite recent advancements, the *in vivo* sensitivity of NanoLuc-derived luciferase-luciferin pairs still falls short compared to FLuc-derived benchmark reporters. This limitation continues to hinder the full potential of NanoLuc-derived bioluminescent indicators. To address this challenge, there is a pressing need for further research to systematically improve the *in vivo* capabilities of NanoLuc-derived luciferases and indicators.

Here, we present a PEGylation method to enhance the auto-oxidation resistance and water solubility of marine luciferase substrates. We showcased the versatility of this approach using three luciferins, resulting in a series of new prosubstrates with remarkably increased solubility. Given the challenges of brain BLI due to the blood-brain barrier (BBB) limiting luciferin entry and the need for capturing rapid neuronal activity,^20^ we focused on characterizing these prosubstrates using mouse models expressing luciferases in the brain. We observed drastic bioluminescence enhancements with these water-soluble luciferins that are deliverable at a safe concentration of 25 mM. Notably, we identified the brightest luciferase-luciferin combination, which surpassed the brightness of Akaluc-AkaLumine pair, allowing high-speed video-rate imaging of freely moving mice with brain-expressed luciferase. Furthermore, we validated the substantial brightness enhancement in nonbrain tissues using one of the novel luciferins and a mouse model with liver luciferase expression. Collectively, these findings strongly indicate that PEGylation of marine luciferase substrates represents a versatile approach to significantly enhance the sensitivity of in vivo BLI.

## Results

### PEGylation of DTZ and Assessment of Its Solubility and Auto-Oxidation Resistance

We previously developed a NanoLuc variant, teLuc, that produced bright bioluminescence peaking around 500 nm when combined with a DTZ substrate (Figure S1A).^21^ More recently, we refined DTZ to create a prosubstrate, ETZ (Figure S1A), which could be administered in higher doses and activated *in vivo* by nonspecific esterase, enhancing *in vivo* bioluminescence.^26^ In parallel, other researchers introduced luciferin variants such as HFz and FFz (Figure S1A), improving water solubility and brightness in nonbrain tissues.^29^ However, the solubility improvements from these prior studies were only modest, and high *in vivo* brightness still required the use of high doses of organic cosolvents or surfactants, which could induce organ toxicity.^20,30^

**Figure 1:**
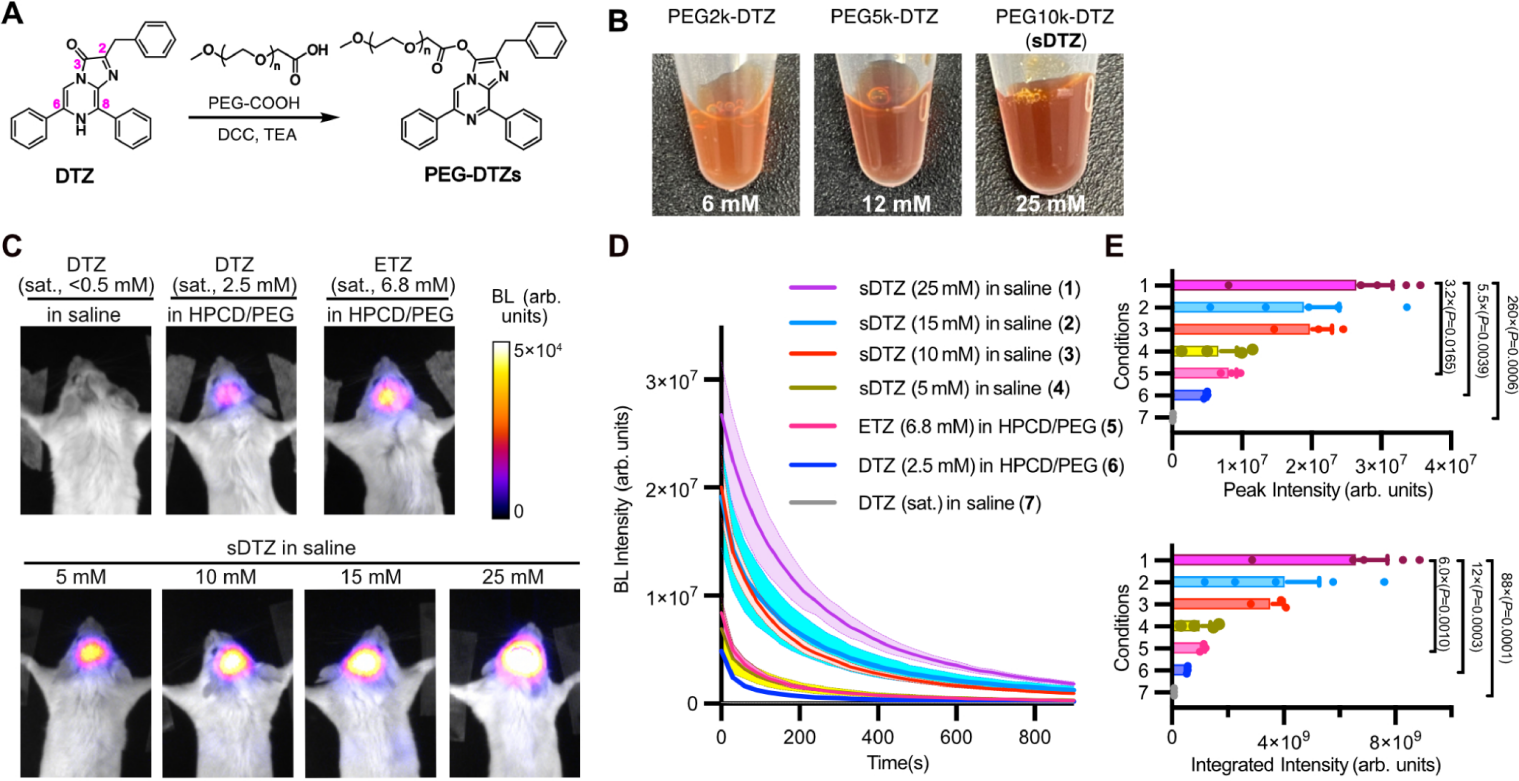
Synthesis of water-soluble DTZ derivatives and evaluation of solubility, stability, and sensitivity for *in vivo* bioluminescence brain imaging. (**A**) Synthetic route to modify DTZ with three lengths of polyethylene glycol (PEG) chains (PEG 2k, 5k, or 10k) via the C3 carbonyl group of DTZ. (**B**) Photos of PEGylated DTZ variants dissolved in normal saline at the indicated near-saturation concentrations. (**C**) Representative bioluminescence images of live mice with hippocampal BREP luciferase expression upon the tail vein delivery of the luciferins at the indicated dose and buffer conditions. Images with peak bioluminescence intensities were presented in pseudocolor overlaid on corresponding brightfield images. (**D**) Quantification of bioluminescence intensity over time for each group in panel C. (**E**) Comparison of peak bioluminescence intensity (top) and bioluminescence integrated over 15 min (bottom). The numbered conditions refer to the groups presented in panel D. *p* values were determined by ordinary one-way ANOVA followed by Dunnett’s multiple comparisons test. Data are presented as mean ± s.e.m. (in panels C-E, *n* = 4 mice for 5 mM sDTZ, *n* = 5 mice for 15 and 25 mM sDTZ, and *n* = 3 mice for other groups).

We aimed to significantly enhance the water solubility of marine luciferase substrates, enabling high-dose delivery via aqueous solutions. Polyethylene glycol (PEG) conjugation is a commonly employed method for improving the solubility of hydrophobic drugs.^31^ Using DTZ as our model compound, we investigated PEGylation at the C3 carbonyl group of the DTZ imidazopyrazine ring (Figures 1A and S1B). We expect PEGylation at this site to create caged substrates, but upon injection into animals, nonspecific esterase would quickly remove the PEGylation, releasing free DTZ luciferin.^26^

By conducting condensation reactions of DTZ with methoxy PEG carboxylic acids of varying molecular weights (∼2000, 5000, and 10 000 Da), we obtained the polymer-luciferin conjugates (PEG2k-DTZ, PEG5k-DTZ, and PEG10k-DTZ) and determined their maximum solubility in normal saline to be approximately 6, 12, and 25 mM, respectively (Figure 1B). Given that PEG10k-DTZ exhibited the highest solubility, we renamed it sDTZ and focused our subsequent experiments on this compound. To provide a basis for comparison, we conducted additional tests to assess the solubility of DTZ in normal saline supplemented with 25 mM PEG10k. The solubility was found to be between 1 and 2 mM (Figure S2A). This observation suggests that the enhanced solubility of sDTZ in normal saline is attributed to the intramolecular ester conjugation with the polymeric PEG chain. Furthermore, we investigated the solubility of sDTZ in mouse serum, and the results indicated a range between 20 and 25 mM (Figure S2B).

CTZ and its analogs have the tendency to undergo slow oxidation in the absence of luciferases, and this oxidation reaction relies on the C3 carbonyl group of the DTZ imidazopyrazine ring.^7^ However, since the C3 position in sDTZ is masked, we anticipate that it will exhibit increased resistance to auto-oxidation. To investigate this, we compared the remaining bioluminescence activity of sDTZ and DTZ compounds under various storage conditions (e.g., as solids at RT and as neutral aqueous solutions at 4 °C, −20 °C, and −80 °C). Higher bioluminescence activity was consistently observed with sDTZ across all tested conditions (Figure S3). Despite the improvement, we still observed auto-oxidation of sDTZ, indicating that the ester linkage is susceptible to hydrolysis by water. This can occur in solution, as well as in the solid state through absorption of atmospheric moisture. We further utilized thin layer chromatography (TLC) to directly examine ester linkage cleavage. The results revealed that in aqueous solution at 4 °C, the hydrolysis of sDTZ commenced within a few hours and was nearly completed in a day (Figure S4).

### Brightness Enhancement for Brain and Liver Imaging, and Toxicity Evaluation

In a prior study, we developed an optimized fusion protein named BREP by combining teLuc with the red fluorescent protein (RFP) mScarlet-I.^26^ BREP, when paired with DTZ, emits about 60% of its total emission above 600 nm, positioning it as a highly effective luciferase for deep-tissue BLI. Replacing DTZ with sDTZ is not anticipated to alter the emission profile (Figure S5), because when sDTZ is administered *in vivo*, it will undergo hydrolysis to convert into DTZ. We generated adeno-associated viruses (AAVs) harboring the BREP gene under the control of the human synapsin I (hSyn) promoter and introduced them into the hippocampal neurons of mice through stereotactic injection. Two to 3 weeks later, the mice with BREP expressed in the brain were examined under anesthesia in a dark box with an EMCCD camera. We conducted a pilot study to evaluate different delivery methods, including intravenous, intraperitoneal, and retro-orbital injections, to administer 100 μL of 25 mM sDTZ solutions (Figure S6). The highest brightness was achieved when the luciferin was delivered via the tail vein. As a result, we utilized tail vein injection in all subsequent studies.

We subsequently evaluated sDTZ for enhancing BLI brightness using the mouse model. Experimenting with four different sDTZ concentrations revealed that higher luciferin concentrations led to increased bioluminescence intensity (Figures 1C,D and S7). The brightest signal occurred with the maximal sDTZ concentration (25 mM) in normal saline. The signal decreased over time and around 7% of the peak intensity was retained after 15 min (Figure 1D). In contrast, DTZ saturated in saline had a peak intensity 260-fold lower and a 15 min integrated intensity 88-fold lower (Figure 1E). Strong bioluminescence was observed when DTZ and ETZ were dissolved in a formulation containing 25% (w/v) 2-hydroxypropyl-β-cyclodextrin (HPCD) and 20% (v/v) PEG-400,^26^ which allowed the maximal final concentrations of DTZ and ETZ to be approximately 2.5 mM and 6.8 mM, respectively. However, the sDTZ in saline group still had a peak intensity 5.5-fold and 3.2-fold higher than DTZ and ETZ, respectively. In addition, the integrated bioluminescence signal over 15 min for the sDTZ in saline group was approximately 12-fold and 6-fold higher than the DTZ and ETZ in HPCD/PEG groups, respectively (Figure 1E). Collectively, the results indicate that sDTZ enables efficient *in vivo* luciferin delivery using normal saline (Table S1), significantly enhancing sensitivity for brain BLI in mice.

**Figure 2:**
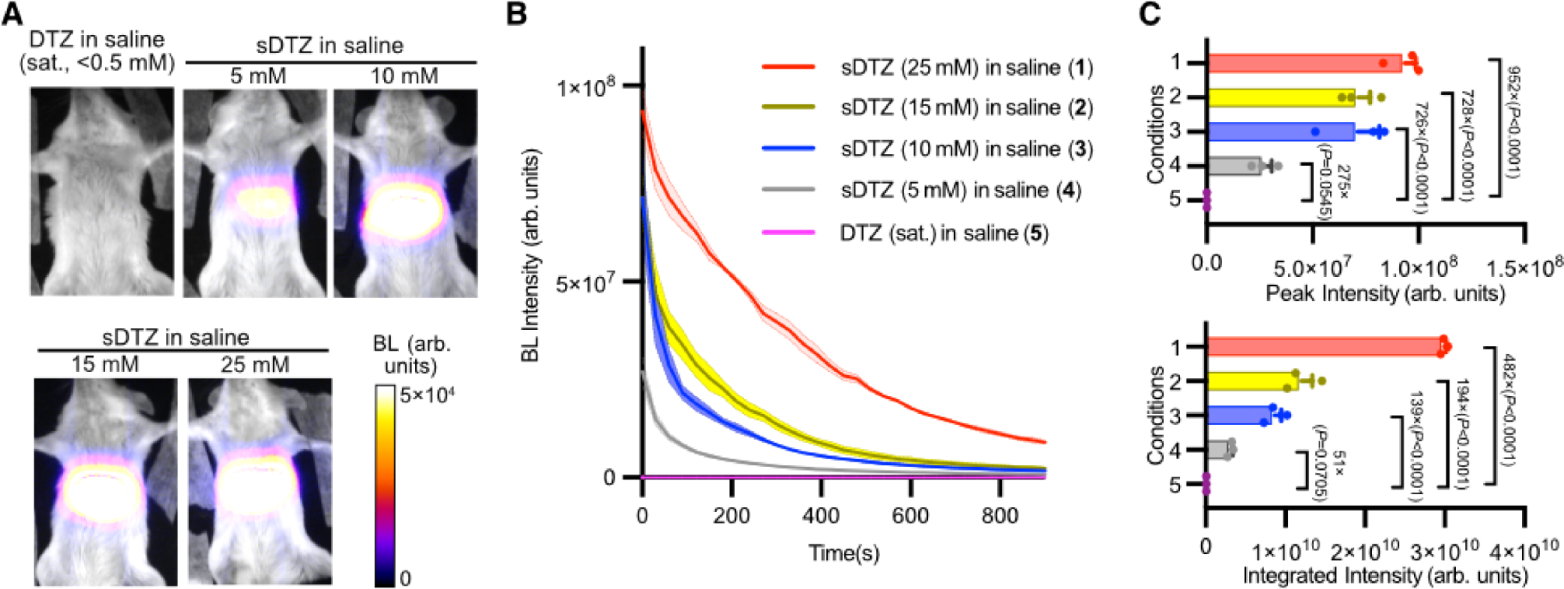
Evaluation of sDTZ paired with BREP for *in vivo* bioluminescence liver imaging. (**A**) Representative bioluminescence images of live mice with liver BREP luciferase expression upon the tail vein delivery of the luciferins at the indicated dose and buffer conditions. Images with peak bioluminescence intensities were presented in pseudocolor overlaid on corresponding bright-field images. (**B**) Quantification of bioluminescence intensity over time for each group in panel A. (**C**) Comparison of peak bioluminescence intensity (top) and bioluminescence integrated over 15 min (bottom). The numbered conditions refer to the groups presented in panel B. *p* values were determined by ordinary one-way ANOVA followed by Tukey’s multiple comparison tests. Data are presented as mean ± s.e.m. (*n* = 3 mice for each group).

To explore bioluminescence improvement in different tissues, we introduced BREP luciferase into mouse livers using AAVs with a hepatocyte-specific thyroxine-binding globulin (TBG) promoter.^23,32^ Mice were administered different concentrations of sDTZ before imaging. The bioluminescence intensity increased with higher sDTZ concentrations, exceeding that of DTZ in saline by at least 2 orders of magnitude (Figures 2 and S8 and Table S1). The 25 mM sDTZ concentration resulted in the highest intensity, with nearly a 1000-fold peak intensity increase and a 482-fold integrated intensity increase over the first 15 min. These findings align with brain imaging results, confirming substantial sensitivity enhancement by sDTZ in other tissue types.

After determining 25 mM sDTZ as optimal for achieving maximum sensitivity, we assessed organ toxicity with five consecutive daily injections. At the end of the experiment, mice were sacrificed, and organs were extracted and examined with hematoxylin and eosin (H&E) staining. No apparent organ toxicity was found, suggesting sDTZ’s safety for animal imaging (Figure S9).

### PEGylation of Alternative Luciferins and Brain Brightness Evaluation

**Figure 3:**
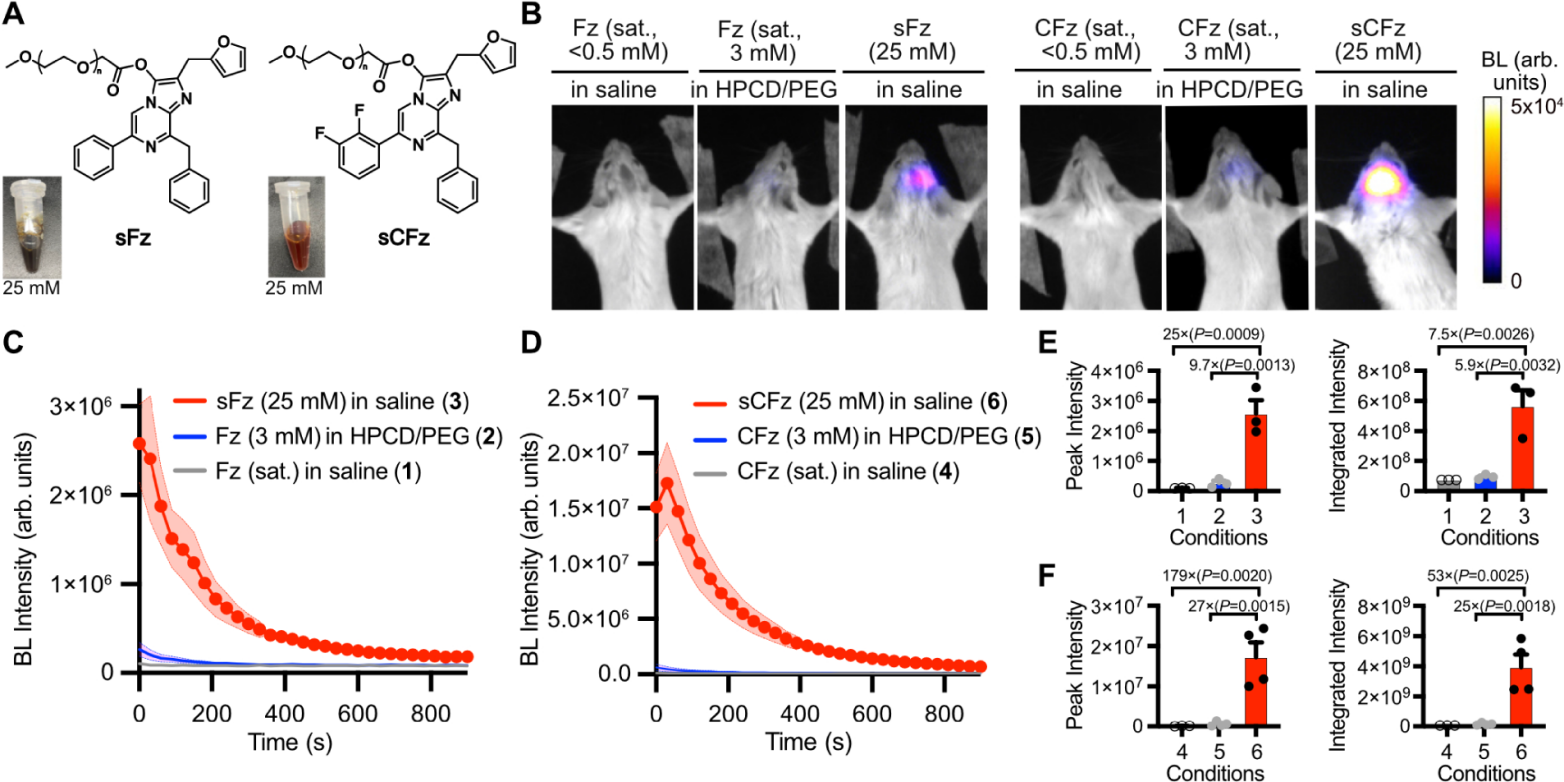
Evaluation of water-soluble Fz and CFz variants, sFz and sCFz, paired with Antares for in vivo bioluminescence brain imaging. (**A**) Chemical structures of sFz and sCFz. PEG10k was used to cage the substrates. Photos of individual substances (25 mM) dissolved in normal saline are also shown. (**B**) Representative bioluminescence images of live mice with hippocampal Antares luciferase expression upon the tail vein delivery of the luciferins at the indicated dose and buffer conditions. Images with peak bioluminescence intensities were presented in pseudocolor overlaid on corresponding brightfield images. (**C**) Quantification of bioluminescence intensity over time for mice in the Fz and sFz groups. (**D**) Quantification of bioluminescence intensity over time for mice in the CFz and sCFz groups. (**E**) Comparison of peak bioluminescence intensity (left) and bioluminescence integrated over time (right) for the Fz and sFz groups. (**F**) Comparison of peak bioluminescence intensity (left) and bioluminescence integrated over time (right) for the CFz and sCFz groups. In panels E and F, the numbered conditions refer to the groups presented in panels C and D; *p* values were determined by ordinary one-way ANOVA followed by Dunnett’s multiple comparisons test. Data are presented as mean ± s.e.m. (in panels B–F, *n* = 4 mice for CFz in HPCD/PEG and sCFz groups, and *n* = 3 mice for other groups).

After achieving success with sDTZ, we extended the PEGylation method to other luciferins. Specifically, we focused on two crucial ones: furimazine (Fz),^17^ the original NanoLuc substrate, and CFz,^20^ a recently developed NanoLuc substrate with improved BBB penetration. We introduced a methoxy PEG chain (MW ∼ 10 000) to create sFz and sCFz, respectively (Figures 3A and S1B). As anticipated, the PEGylation of Fz and CFz increased their resistance to auto-oxidation (Figure S3). To assess sFz and sCFz for *in vivo* BLI, we used AAVs to express the Antares luciferase,^24^ a fusion of NanoLuc to two copies of CyOFP1, in the hippocampal region of the mouse brain. We injected sFz or sCFz (25 mM in normal saline) via the tail vein and compared results with Fz or CFz saturated in the HPCD/PEG buffer mentioned earlier, as well as normal saline. Both sFz and sCFz significantly enhanced brightness (Figures 3B**–**F and S10). Particularly, sCFz led to a 179-fold and 27-fold peak bioluminescence increase compared to CFz in normal saline and the HPCD/PEG buffer, respectively. Moreover, the sCFz group showed approximately 5–6-fold brighter signals than sFz (Figure 3E,F), consistent with CFz’s better BBB permeability than Fz.^20^ These findings indicate that PEGylation can improve solubility and greatly enhance *in vivo* BLI sensitivity across different luciferins.

### Identification of the Optimal Luciferase-Luciferin Combination and High-Speed Video-Rate Brain Imaging of Freely Moving Mice

**Figure 4:**
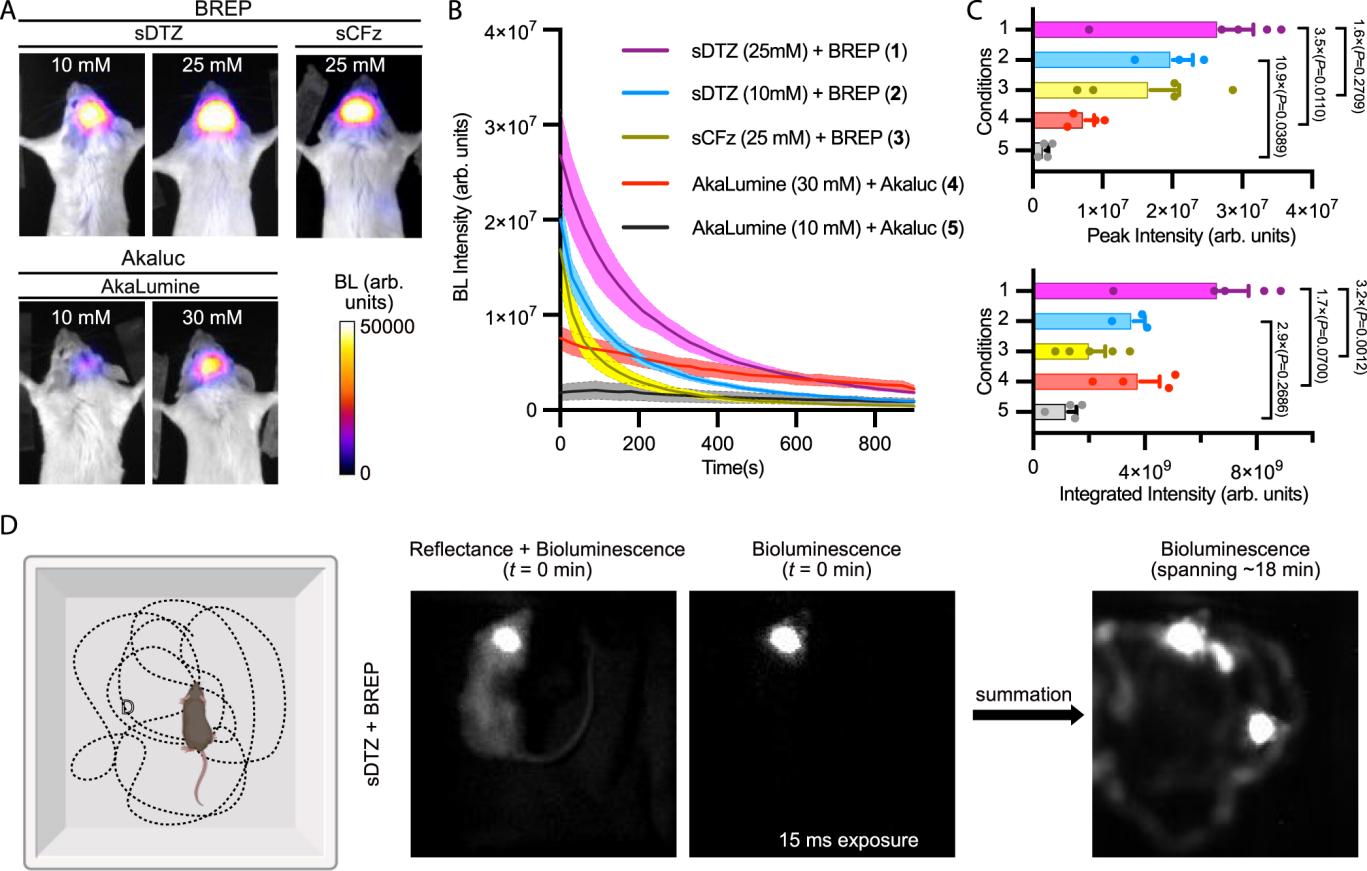
Further brightness comparison and high-frame video-rate *in vivo* bioluminescence brain imaging using BREP and sDTZ. (**A**) Representative bioluminescence images of live mice with hippocampal luciferase expression upon the tail vein delivery of the indicated luciferins at the indicated dose using normal saline. Comparisons were made between BREP (paired with sDTZ or sCFz) and Akaluc (paired with Akalumine). Images with peak bioluminescence intensities were presented in pseudocolor overlaid on corresponding brightfield images. (**B**) Quantification of bioluminescence intensity over time among different groups. (**C**) Comparison of peak bioluminescence intensity (top) and bioluminescence integrated over time (bottom). The numbered conditions refer to the groups presented in panel B. *p* values were determined by ordinary one-way ANOVA followed by Tukey’s multiple comparison tests. Data are presented as mean values ± s.e.m. (*n* = 3 mice for BREP and 10 mM sDTZ, *n* = 4 mice for Akaluc and 10 or 30 mM AkaLumine, and *n* = 5 mice for all other groups). The same data for BREP and sDTZ in panels A-C are also presented in Figure 1C–E. (**D**) High frame-rate imaging of a mouse with hippocampal BREP luciferase expression. The mouse was injected with sDTZ via the tail vein right before imaging. Brightfield and bioluminescent images (15 ms exposure time for each) were alternately acquired using an EMCCD camera. The first frame (at *t* = 0 min) was shown. A series of 280 frames of bioluminescent images at each time point (*t* = 0, 3, 6, 12, and 18 min) were acquired, and the total 1,400 frames were combined to create the summation image. Results for the same experiments are also presented in Figure S12.

Based on our data, both BREP-sDTZ and Antares-sCFz combinations displayed bright bioluminescence, with BREP-sDTZ being roughly twice as bright as Antares-sCFz (Figures 1E and 3F). Considering the cross-reactivity of teLuc in BREP toward NanoLuc substrates (Figure S5), we further examined the bioluminescence of mice with hippocampal BREP expression after delivering sCFz (Figures 4A and S10). The BREP-sCFz mice exhibited strong bioluminescence, with intensity below the BREP-sDTZ mice (Figure 4B,C). Although the sample size was inadequate to statistically distinguish the peak intensities, BREP-sCFz displayed a quicker bioluminescence decay and lower 15 min integrated bioluminescence that is statistically significant compared to BREP-sDTZ (Figure 4B,C and Table S1). These findings indicate that the BREP-sDTZ combination exhibits the most promise among the tested ATP-independent luciferase-luciferin pairs.

In addition, we compared BREP-sDTZ with the ATP-dependent luciferase-luciferin pair, Akaluc-AkaLumine.^15^ All luciferins were administered intravenously in normal saline to mice with hippocampal luciferase expression. At 10 mM luciferin concentrations, the peak intensities of BREP mice were approximately 11 times brighter than Akaluc mice (Figures 4A–C and S11). With 25 mM sDTZ and 30 mM AkaLumine, BREP remained 3.5 times brighter than Akaluc. Despite displaying a faster bioluminescence decay, BREP-sDTZ consistently exhibited greater brightness in the initial 10 min, indicating its suitability for imaging applications demanding high sensitivity (Figure 4B).

Given the excellent performance of sDTZ, we conducted high-speed video-rate BLI of freely moving mice expressing BREP in the hippocampus (Figures 4D and S12, and Movie S1). The bioluminescence glow from the brain was so bright and even visible in brightfield imaging at the beginning of the experiment. By alternating between brightfield and bioluminescence imaging, we successfully captured mouse movement and brain bioluminescence for over 18 min using a 15 ms exposure. Previous research has demonstrated the capability to monitor Akaluc and Antares signals in moving mouse brains using AkaLumine or CFz.^15,20^ Despite variations in protocols and equipment, our study achieved enhanced temporal resolution by utilizing BREP-sDTZ, even in unshaved mice.

## Discussion

The primary contribution of this work is the development of a PEGylation method for modifying marine luciferase substrates. By attaching PEG chains to these substrates, their water-solubility was greatly enhanced, allowing for effective *in vivo* luciferin administration using a simple and nontoxic normal saline solution. The resulting luciferase-luciferin pairs exhibited notable increases in brightness, surpassing established benchmarks such as Akaluc-AkaLumine and Antares-CFz.^15,20,24^ This research tackles critical obstacles in the field, achieving optimal sensitivity with ATP-independent luciferase reporters and enabling high-speed video-rate imaging of freely moving animals.

It is worth noting that the PEGylated substrates are expected to undergo rapid decaging upon injection into animals, similar to our previous study on ETZ which confirmed the labile nature of esterification through the C3 carbonyl group *in vivo*.^26^ The presence of PEG chains is not anticipated to affect BBB crossing, except for enabling the delivery of higher doses in aqueous buffers. Additionally, the elimination of organic cosolvents or surfactants from the luciferin delivery solution enhances the biocompatibility of BLI. Previous studies have often utilized Poloxamer 407 (*p*-407) as a delivery vehicle for similar luciferins, but multiple injections have been shown to induce liver, kidney, and heart toxicity, atherosclerosis, and weight loss in mice.^20,30^

Furthermore, the extension of the PEGylation technology to multiple luciferins signifies a significant advancement. CFz has recently been reported as a promising luciferin for brain imaging.^20^ In our research, we achieved a remarkable 179-fold and 27-fold increase in bioluminescence brightness using sCFz compared to CFz saturated in normal saline or an HPCD/PEG buffer (Figure 3). This study highlights the versatility of PEGylation with various CTZ analogs, expanding the potential applications and research opportunities in this field.

These PEGylated ATP-independent luciferins demonstrated increased resistance to auto-oxidation compared to their parental counterparts (Figure S3), due to the presence of a caged carbonyl group that is essential for the oxidation reaction.^7^ However, it is crucial to acknowledge that the ester linkage in these PEGylated luciferins is relatively labile, as evidenced by hydrolysis observed both *in vitro* and *in vivo*. While rapid *in vivo* hydrolysis is advantageous for bioluminescence, *in vitro* hydrolysis can lead to substrate auto-oxidation and degradation before use. For long-term storage, we recommend storing these PEGylated luciferins in the solid format at temperatures of −20 °C or −80 °C. Prior to conducting experiments, it is important to dissolve the solids in a neutral aqueous solution, as the presence of base or acid can catalyze ester hydrolysis. Once the solution is prepared, it remains usable for only a few hours on ice or a few days at −20 °C or −80 °C (Figures S3 and S4). In contrast, ATP-dependent luciferins such as AkaLumine exhibit greater chemical stability, as they remain inactive until activated by ATP. This distinction is important to consider in terms of handling and storage requirements for these two categories of luciferins.

Our animal validation experiments were centered on brain imaging, considering the challenges posed by the BBB and the essential requirement for high temporal resolution to capture rapid neuronal activities. Compared to the Akaluc-AkaLumine pair,^15^ we here gained 3.5- to 10.9-fold bioluminescence brightness increase using ATP-independent BREP-sDTZ (Figure 4C). The discovery of BREP-sDTZ as the most luminous luciferase-luciferin combination opens up new possibilities for accurate and sensitive imaging of targets in live organisms. The advantages of BREP-sDTZ would be particularly notable in scenarios where the detection of a small number of cells is beneficial, such as in developmental biology and cancer metastasis research. Furthermore, by combining these new luciferins with rapidly expanding bioluminescent biosensors,^25,27,33−37^ our study provides a pathway for noninvasively capturing fast biological dynamics, including neuronal activity. Additionally, ATP-independent bioluminescence systems described here offer the benefit of minimal disruption to biological processes, as ATP is an endogenous energy and signaling molecule.^38^ These systems also exhibit effective functionality in extracellular spaces and biological fluids like serum and urine, where ATP concentrations are typically low.^16^ Conversely, Akaluc-AkaLumine relies on ATP for bioluminescence and does not function effectively under these conditions.^16^

When it comes to generating sustained signals, Akaluc-AkaLumine continues to outperform ATP-independent bioluminescence systems, including BREP-sDTZ (Figure 4B and Table S1). Further research is necessary to address this limitation and continue making progress from the work presented here. Additionally, we can envision the fast signal decay of BREP-sDTZ becoming an advantage under certain circumstances. For example, because it is often challenging to spectrally separate bioluminescent reporters *in vivo*, BLI with multiple luciferases may require sequential injection and imaging using orthogonal luciferins. Since the signals of BREP-sDTZ would largely dissipate after 1–2 h following luciferin delivery, it would allow quicker imaging of the second reporter.

In this study, luciferins were delivered into mice via tail vein injection, as our main objective was to achieve the highest peak bioluminescence intensity. Compared to intraperitoneal injection, tail vein injection yielded approximately 100-fold higher bioluminescence, albeit with a shorter signal duration (Figure S6). Moreover, retro-orbital injection demonstrated promising results, as it produced bright bioluminescence and appeared to have a longer signal duration compared to tail vein injection. We intend to further investigate and explore this approach in our future studies.

This study further illustrates that the application of PEGylated luciferins extends to nonbrain tissues. The use of sDTZ allowed for high-sensitivity BLI of the liver, indicating that the novel tools may facilitate the study of various organ-specific processes, disease models, and therapeutic interventions in nonbrain systems.

In summary, this work has introduced a PEGylation method to enhance a range of marine luciferase substrates, achieving the highest bioluminescence sensitivity with these novel caged PEGylated luciferins. These new tools provide researchers with unparalleled sensitivity, resolution, and versatility, serving as invaluable assets for furthering our comprehension of biological processes and disease mechanisms.

## Methods

### Ethical Statement

All animal studies were carried out per the University of Virginia Institutional Animal Care and Use Committee’s approval (Protocol #4196) and guidelines. BALB/cJ mice (#000651) and C57BL/6 J mice (#000664) were procured from the Jackson Laboratory and bred under standard conditions. The mice were housed in a temperature-controlled environment (∼23 °C) with a 12 h/12 h light-dark cycle and ∼50% humidity. Animals were randomly allocated to experimental groups to ensure a mix of male and female subjects.

### Chemical Synthesis and Characterization

Methoxy PEG acetic acids (PEG-COOH) with molecular weights of ∼2000 Da (Cat. # A3138), ∼5000 Da (Cat. # A3071), and ∼10 000 Da (Cat. # A3081) were procured from JenKem Technology. DTZ, Fz, and CFz were synthesized following established procedures,^17,20,21^ and purified using a Waters Prep 150/SQ Detector 2 LC-MS Purification System with MassLynx software (Version 4.2) and an XBridge BEH Amide/Phenyl OBD Prep Column (130 Å, 5 μm, 30 mm × 150 mm). For the conjugation reaction, methoxy PEG acetic acid (0.02 mmol, 1 equiv) and DCC (12.4 mg, 0.06 mmol, 3 equiv) were combined in a dried and argon-purged two-neck 50 mL round-bottom flask. A mixture of anhydrous dichloromethane (10 mL) and triethylamine (4.4 μL, 0.06 mmol, 3 equiv) was slowly added while stirring at RT for 20 min. Then, DTZ, Fz, or CFz (0.024 mmol, 1.2 equiv) was swiftly introduced into the reaction mixture. The reaction was stirred under argon for an additional hour and monitored by TLC (DCM/methanol = 10:1). Upon completion, the reaction mixture was concentrated *in vacuo*, and the residue was washed with ethyl ether and purified by silica gel chromatography (DCM/methanol = 15:1, v/v). The resulting compound was collected, concentrated, dissolved in 5 mL ddH_2_O, filtered through VWR grade 415 qualitative filter paper, and lyophilized to obtain sDTZ, sFz, or sCFz as solids using a 12-port Labconco freeze-dryer with an Edwards RV3 vacuum pump. The purity of the compounds was reverified using TLC (Figure S13), and the recovery yields based on the input of PEG-COOH were approximately 80%. ^1^H NMR spectra were recorded on a Bruker Avance III 600 MHz NMR spectrometer with Bruker TopSpin IconNMR software (Version 3.5pl4) and analyzed using MestReNova software (Version 12.0.3). Figures S14 to S17 display the ^1^H NMR spectra for DTZ, Fz and CFz (dissolved in Methanol-*d*_4_), as well as sDTZ, sFz, sCFz and the starting material, PEG10k (dissolved in D_2_O). **DTZ:**^1^H NMR (600 MHz, methanol-*d*_4_) δ 8.10–8.01 (m, 2H), 7.95–7.65 (m, 3H), 7.64–7.54 (m, 3H), 7.49 (t, *J* = 7.5 Hz, 2H), 7.46–7.38 (m, 1H), 7.38–7.21 (m, 4H), 7.15 (t, *J* = 7.4 Hz, 1H), 4.16 (s, 2H). **sDTZ:**^1^H NMR (600 MHz, D_2_O) δ 8.39 (s, 1H), 8.05–8.00 (m, 3H), 7.64–7.53 (m, 4H), 7.14–6.98 (m, 8H), 4.01 (s, 2H), 3.82 (s, 2H), 3.51–3.40 (m, PEG), 3.18 (s, 3H). **Fz:**^1^H NMR (600 MHz, methanol-*d*_4_) δ 7.77 (s, 1H), 7.68 (d, *J* = 7.4 Hz, 2H), 7.51–7.44 (m, 3H), 7.43–7.40 (m, 2H), 7.38 (dd, *J* = 1.9, 0.9 Hz, 1H), 7.33–7.27 (m, 2H), 7.25–7.21 (m, 1H), 6.32 (s, 1H), 6.10 (s, 1H), 4.43 (s, 2H), 4.19 (s, 2H). **sFz:**^1^H NMR (600 MHz, D_2_O) δ 8.63 (s, 1H), 8.43–8.19 (m, 1H), 8.10 (s, 1H), 7.85–7.80 (m, 3H), 7.50–7.28 (m, 9H), 6.39 (s, 1H), 6.23 (s, 1H), 4.64 (s, 2H), 4.20 (m, 2H), 4.03 (s, 2H), 3.65–3.59 (m, PEG), 3.30 (s, 3H). **CFz:**^1^H NMR (600 MHz, methanol-*d*_4_) δ 7.87 (s, 1H), 7.53 (s, 1H), 7.44–7.35 (m, 3H), 7.34–7.26 (m, 3H), 7.23 (t, *J* = 7.5 Hz, 1H), 6.32 (s, 1H), 6.10 (s, 1H), 4.41 (s, 2H), 4.19 (s, 2H). **sCFz:**^1^H NMR (600 MHz, D_2_O) δ 8.62 (s, 1H), 8.43–8.37 (m, 2H), 8.10 (s, 1H), 7.47–7.08 (m, 6H), 6.37 (s, 1H), 6.22 (s, 1H), 4.64 (s, 2H), 4.18 (m, 2H), 4.00 (s, 2H), 3.65–3.58 (m, PEG), 3.29 (s, 3H). **PEG10k:**^1^H NMR (600 MHz, D_2_O) δ 4.10 (s, 2H), 3.66–3.54 (m, PEG), 3.30 (s, 3H).

### Evaluation of Auto-Oxidation Resistance

DTZ and sDTZ were evaluated in two forms: as 100 μM solutions in Tris-HCl buffer (100 mM, pH 7.4) at temperatures of 4, −20, and −80 °C, and as powders stored at RT in aluminum foil-wrapped microcentrifuge tubes to shield from light exposure. The remaining bioluminescence activity was assessed using purified BREP luciferase. For the assays, solid powders were dissolved in Tris-HCl buffer (100 mM, pH 7.4) to obtain 100 μM solutions. DTZ solutions were directly used, while sDTZ solutions underwent pretreatment with porcine liver esterase (MilliporeSigma, Cat. # E3019) by incubating 100 μL of the sDTZ solution with 1 μL of 50 units/mL enzyme for 15 min at RT. Subsequently, DTZ or esterase-treated sDTZ were mixed with purified BREP protein to achieve final concentrations of 50 μM luciferin and 10 nM enzyme. Bioluminescence spectra were recorded using a BMG Labtech CLARIOstar Plus microplate reader controlled by BMG Labtech Reader Software (Version 5.70 R2), with results automatically exported to BMG Labtech MARS Data Analysis Software (Version 3.42 R5). A similar procedure was employed to compare sFz and sCFz with Fz and CFz as 100 μM solutions in Tris-HCl buffer (100 mM, pH 7.4) at 4 °C

### Toxicity Assessment in Mice

sDTZ (25 mM) was dissolved in normal saline (0.9% NaCl, Baxter, Cat. # G158220), and a daily intraperitoneal injection of 100 μL of the solution was administered to C57BL/6J mice for five consecutive days. Normal saline without other compounds served as the control. Tissues from the brain, heart, liver, lung, kidney, and spleen of the mice were harvested at the end of the fifth day. The UVA Research Histology Core Facility conducted paraffin-embedded tissue sectioning and H&E staining.

### BLI of Mice with Hippocampal Luciferases

The process of generating AAVs for neuronal expression of BREP has been detailed previously.^26^ To create analogous viral vectors for expressing Akaluc or Antares, the BREP gene in the pAAV-hSyn-BREP transfer plasmid was substituted with the Akaluc or Antares gene, resulting in the pAAV-hSyn-Akaluc or pAAV-hSyn-Antares transfer plasmid for viral packaging. The titers of the purified viral stocks were quantified using qPCR and were subsequently diluted with Dulbecco’s Phosphate-Buffered Saline (DPBS) to achieve concentrations of 10^14^ GC/mL before use. One μL of the diluted virus was bilaterally injected into the hippocampus (relative to Bregma: AP −1.7, ML ± 1.2 and DV −1.5) of 8-week-old BALB/cJ mice via intracranial stereotactic injection at a flow rate of 100 nL/min. Mice were imaged two to 3 weeks later. A procedure described previously^26^ was employed to dissolve DTZ or ETZ in a buffer comprising 25% (w/v) HPCD and 20% (v/v) PEG-400, and 5% (w/v) NaHCO_3_ was additionally added to facilitate the dissolution of ETZ. Alternatively, the luciferins were simply dissolved in normal saline. Right before imaging, mice received a 100 μL intravenous injection of the dissolved luciferins. They were subsequently imaged using a UVP BioSpectrum dark box, a Computar Motorized ZOOM lens (M6Z1212MP3), and a Photometrics Evolve 16 EMCCD camera. The lens adjustments were made through the UVP VisionWorksLS software (Version 8.6), with the aperture set to 100% open, zoom at 0%, and focus to 0%. Micro-Manager (Version 2.0.0) was utilized to control the camera for imaging acquisition. The camera analog gain was set to high, and the EM gain was 500. Other parameters included a camera binning of 2 × 2, camera temperature maintained at −70 °C, and exposure time of 100 ms with acquisitions every 30 s. Mice were anesthetized and positioned 23 cm away from the lens without an emission filter. The images were processed using the Fiji version of ImageJ 2.14. Initial background subtraction from the image stacks was conducted with a rolling ball radius set to 100 pixels. Subsequently, a region of interest (ROI) was defined based on the bioluminescence signal from the mouse brain, and the integrated intensity value over the ROI was extracted for further analysis. Despite software-based background subtraction, residual background was noted in the images. To address this, the ROI was relocated away from the mouse brain region to evaluate the residual background, which was then subtracted from the signals to calculate the integrated bioluminescence intensity (area under the curve). The data were plotted and subjected to statistical analysis using GraphPad Prism (Version 8.4.3).

### BLI of Mice with Liver BREP Luciferase

To evaluate sDTZ for deep-tissue imaging in the liver, we developed a pAAV-TBG-BREP transfer plasmid featuring a liver-specific promoter. The AAVs were produced and purified following established protocols.^26^ Subsequently, we administered 100 μL (approximately 1 × 10^11^ GC/mL) of AAV-TBG-BREP to mice via tail vein injection. After a 3-week period, the mice were subjected to brightness assessment. Luciferins were dissolved in saline and administered intravenously. All parameters for BLI were consistent with those outlined in the previous section.

### BLI of Freely Moving Mice

BALB/cJ mice expressing hippocampal BREP were prepared following the procedure described above. Before imaging, 100 μL of 25 mM sDTZ was intravenously injected into awake mice. The mice were then positioned in the dark box for BLI. The imaging setup was similar to the one described above, with the addition of a TTL device to regulate the camera and an 850 nm LED light source. During each imaging cycle, the LED and camera were activated for 15 ms to capture a brightfield image, immediately followed by a 15 ms frame of bioluminescence with the camera on and LED off. The camera binning and EM gain were adjusted to 8 × 8 and 1000, respectively. Other parameters remained consistent with the settings detailed above. As large data sets were derived and the system was unable to sustain continuous generation of TTL pulses beyond 8.4 s, we recorded separate 8.4-s videos at time intervals of 0, 3, 6, 12, and 18 min.

## Supporting Information Available

Chemical synthesis and characterization, animal toxicity study, bioluminescence spectra, and additional imaging data (PDF)
Movie S1: High frame-rate brightfield and bioluminescence imaging of a mouse with hippocampal BREP luciferase expression for over 18 min (MP4)

## Abbreviations

AAV: adeno-associated virus
Arb. units: arbitrary units
BBB: blood-brain barrier
BL: bioluminescence
BLI: bioluminescence imaging
CTZ: coelenterazine
FP: fluorescent protein
PEG: polyethylene glycol
Sat.: saturated
