# Supplementary material for: PEGylated ATP-Independent Luciferins for Noninvasive High-Sensitivity High-Speed Bioluminescence Imaging
Source: ACS Chem Biol. 2024 Dec 23;20(1):128–36. doi: 10.1021/acschembio.4c00601 (PMC11744661; doi:10.1021/acschembio.4c00601)
Supplement: Supplementary file 1 — cb4c00601_si_001.pdf [file cb4c00601_si_001.pdf]

Supporting Information

PEGylated ATP-Independent Luciferins for Non-Invasive High-Sensitivity High-Speed Bioluminescence Imaging

Xiaodong Tian,[1,2‡] Yiyu Zhang,[1,2‡] and Hui-wang Ai[1,2,3*]

[1] Department of Molecular Physiology and Biological Physics, University of Virginia School of Medicine, Charlottesville, Virginia 22908, USA.

[2] Center for Membrane and Cell Physiology, University of Virginia School of Medicine, Charlottesville, Virginia 22908, USA.

[3] The UVA Comprehensive Cancer Center, University of Virginia, Charlottesville, Virginia 22908, USA.

[‡]These two authors contributed equally to this work.

*Corresponding author. Email: huiwang.ai@virginia.edu

A

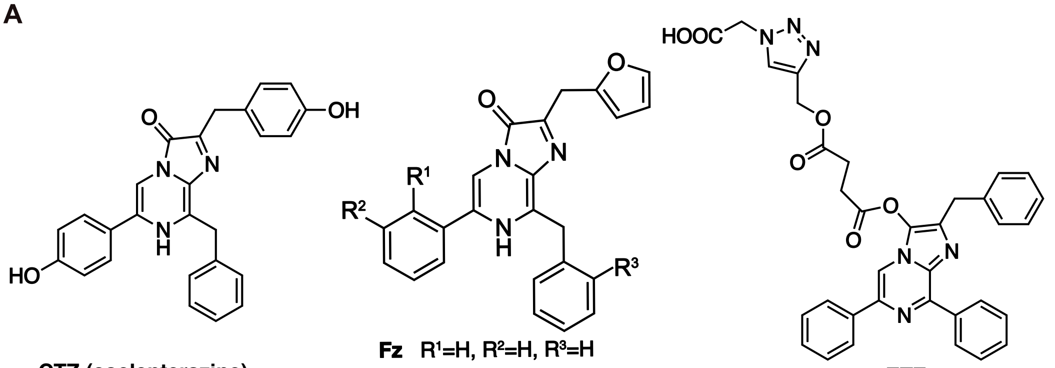

CTZ (coelenterazine)

Fz R¹=H, R²=H, R³=H
FFz R¹=F, R²=NH_2, R³=F
HFz R¹=H, R²=OH, R³=F

ETZ

B

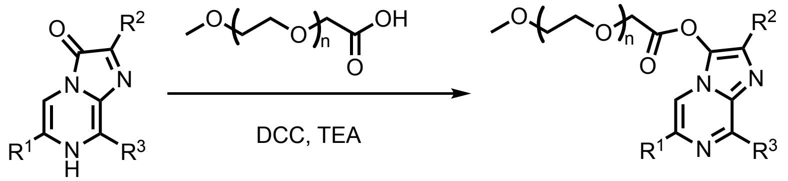

R¹	R²	R³	PEG MW	Reactant	Product
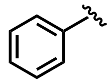	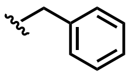	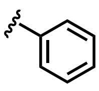	~2 kDa, ~5 kDa, or ~10 kDa	DTZ	PEG2k-DTZ, PEG5k-DTZ, PEG10k-DTZ (sDTZ)
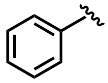	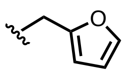	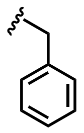	~10 kDa	Fz	sFz
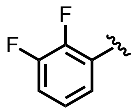	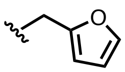	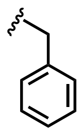	~10 kDa	CFz	sCFz

Fig. S1. Chemical structures of the indicated luciferins (**A**) and the general synthesis route for different water-soluble luciferins (**B**). The detailed synthetic procedures are outlined in the Materials and Methods section.

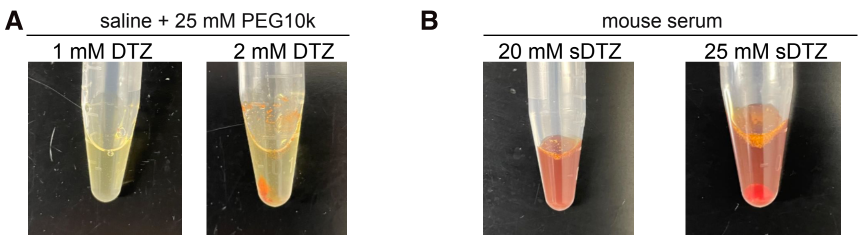

Fig. S2. (**A**) Solubility test revealed that the solubility of DTZ in normal saline supplemented with 25 mM PEG10k ranged between 1 and 2 mM. (**B**) Solubility test demonstrated that the solubility of sDTZ in mouse serum ranged between 20 and 25 mM.

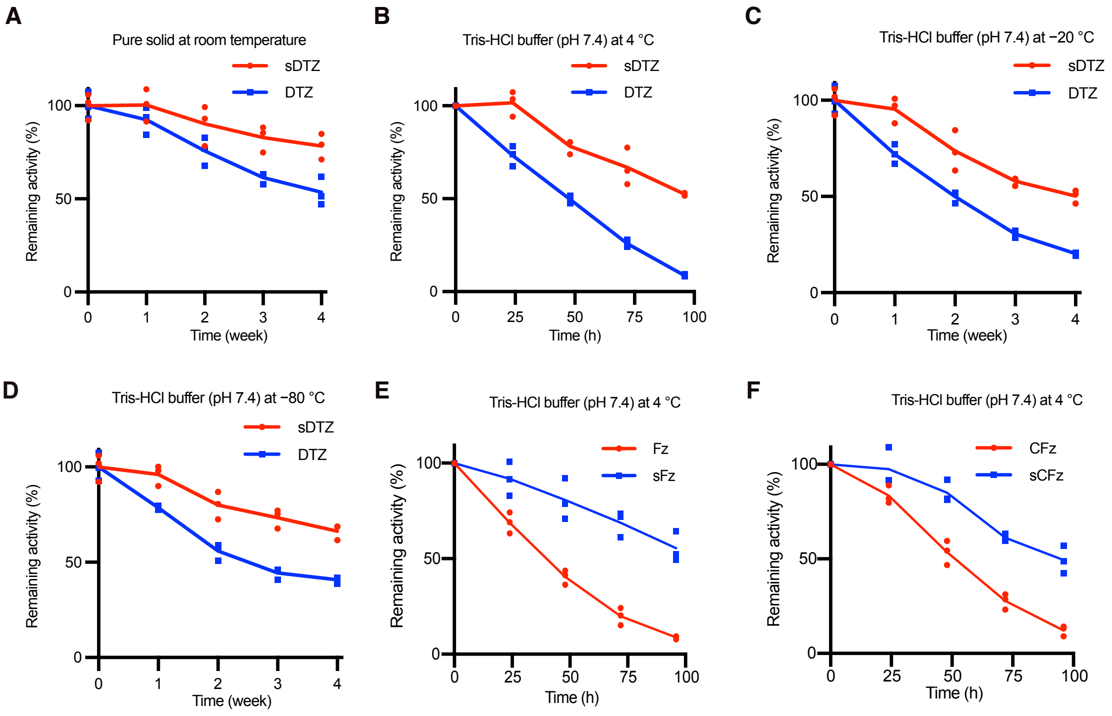

Fig. S3. Comparison of PEGylated and unPEGylated luciferins under the indicated conditions for the retention of bioluminescence activities. (**A**) DTZ and sDTZ in the solid state at room temperature. (**B-D**) DTZ and sDTZ in neutral aqueous buffer (100 mM Tris-HCl, pH 7.4) at 4 °C (**B**), −20 °C (**C**) and −80 °C (**D**). (**EF**) Fz and sFz (**E**), or CFz and sCFz (**F**) in neutral aqueous buffer (100 mM Tris-HCl, pH 7.4) at 4 °C. n= 3 technical repeats. The instability of the PEGylated luciferins was attributed to the hydrolysis of the PEGylation and the subsequent oxidation by oxygen in the air. These experiments may potentially underestimate luciferin degradation at early time points due to the possible saturation of enzymes at high substrate concentrations.

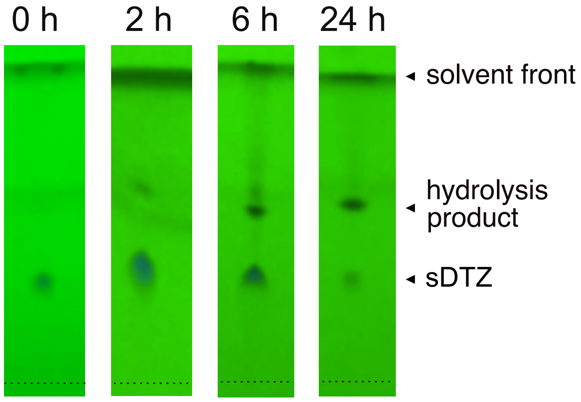

Fig. S4. TLC (DCM/methanol = 10:1) to examine the stability of the ester bond in sDTZ at 4 °C in normal saline (pH ~7.2) over the indicated incubation periods. Prior to spotting the compounds on the TLC plates, DCM extraction from the aqueous phase was conducted at room temperature. This extraction step may lead to overestimating bond cleavage, as the hydrolyzed product is more likely to partition into the organic phase.

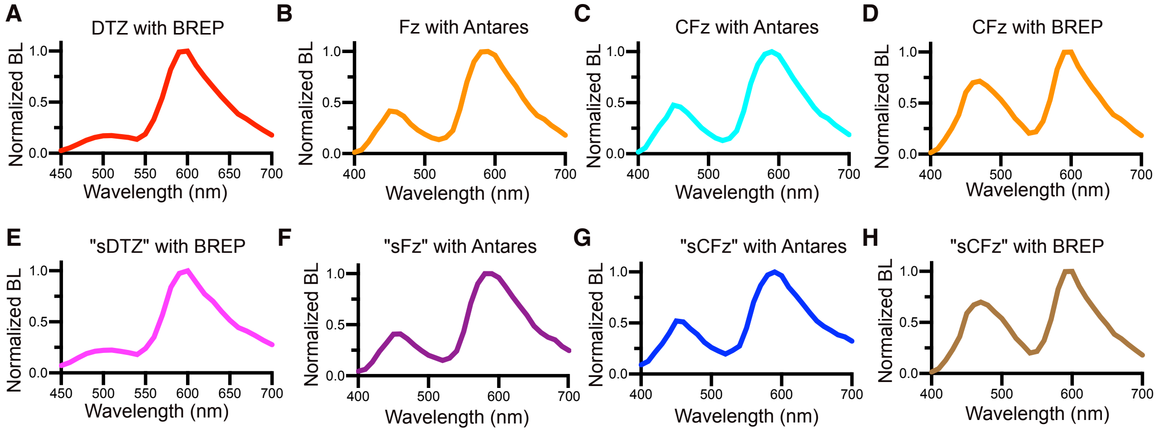

Fig. S5. Normalized bioluminescence emission spectra for the indicated luciferase and luciferin combinations. The spectra were recorded using a 40-fold dilution of cell lysates from bacteria expressing BREP or Antares. The dilution buffer was 100 mM Tris-HCl (pH 7.4), and the final luciferin concentration was 25 μM. In panels E-H, the bioluminescence observed was attributed to the reactions between the luciferases and DTZ, Fz, or CFz derived from the autohydrolysis of sDTZ, sFz, and sCFz; the intensities were much lower compared to those in panels A-D. The spectra were individually normalized to showcase their respective shape.

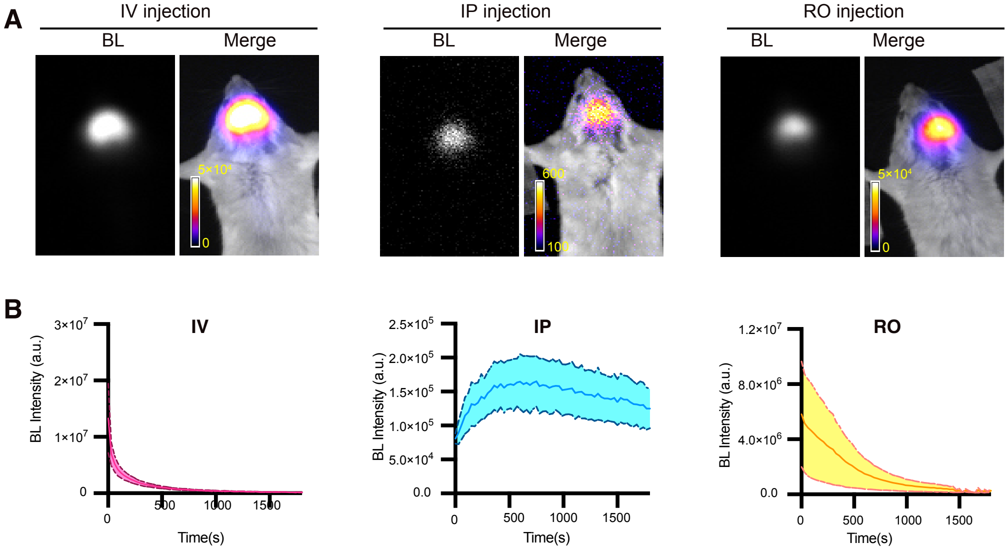

Fig. S6. Pilot study examining different luciferin delivery routes for brain bioluminescence intensities. (**A**) Grayscale and pseudocolored bioluminescence images of live mice with hippocampal BREP luciferase expression. The peak bioluminescence intensities for each luciferin delivery route were displayed. 25 mM sDTZ in normal saline was delivered using the indicated routes. (**B**) Quantification of bioluminescence intensity over time for each injection model. Data are presented as mean ± s.e.m. (n = 3 mice for each group). IV, intravenous; IP, intraperitoneal, RO, retro-orbital.

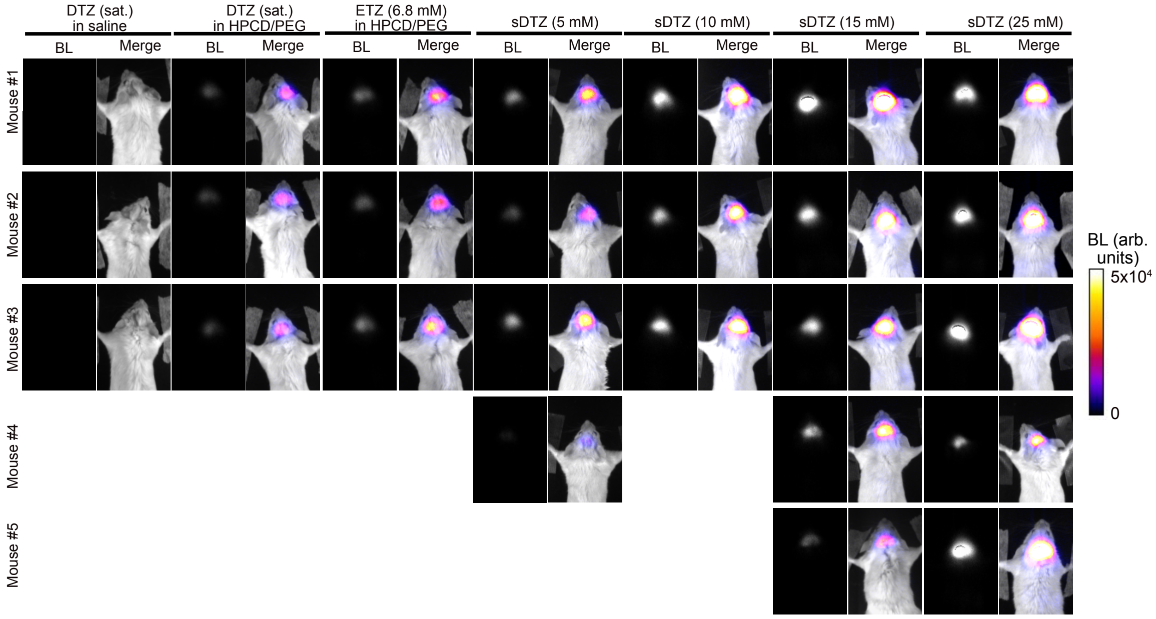

Fig. S7. Comparison of synthetic luciferins (DTZ, ETZ, and sDTZ) for brain imaging in mice. Live mice with hippocampal BREP AAV transduction were subjected to BLI. Substrates (100 µL) in the indicated injection buffers at specified concentrations were administered via the tail vein. Normal saline was used as the delivery solvent for all sDTZ experiments. Grayscale images (left) and pseudocolored images overlaid on corresponding brightfield images (right), displaying peak BL intensities from each substrate injection, are presented. A subset of these images is also featured in Figs. 1C and 4A.

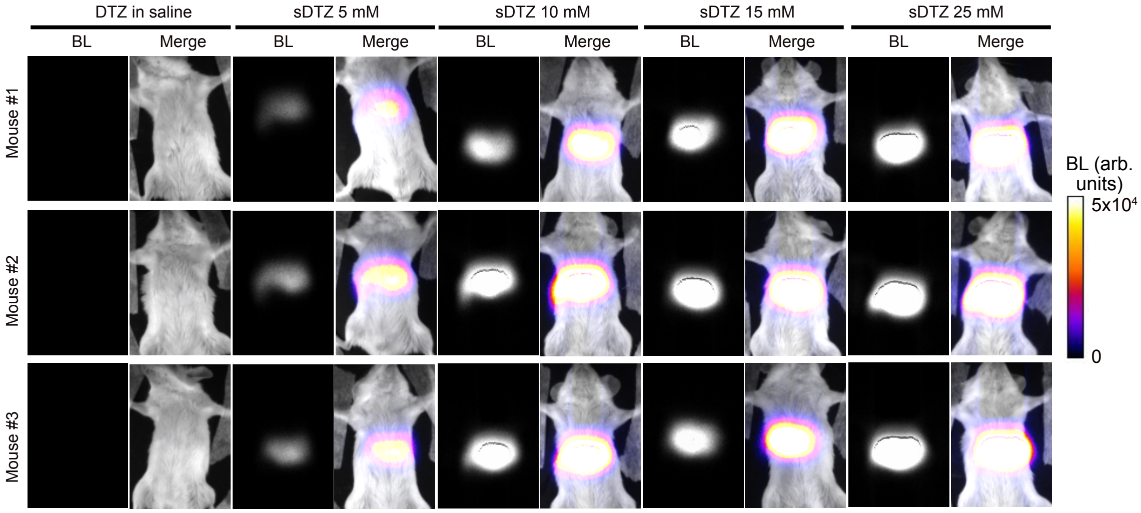

Fig. S8. Comparison of synthetic luciferins (DTZ and sDTZ) for liver imaging in mice. Live mice with liver BREP AAV transduction were subjected to BLI. Substrates (100 µL) in normal saline at specified concentrations were administered via the tail vein. Grayscale images (left) and pseudocolored images overlaid on corresponding brightfield images (right) are used to present peak BL intensities from each substrate injection. A group of these images are also shown in Fig. 2A.

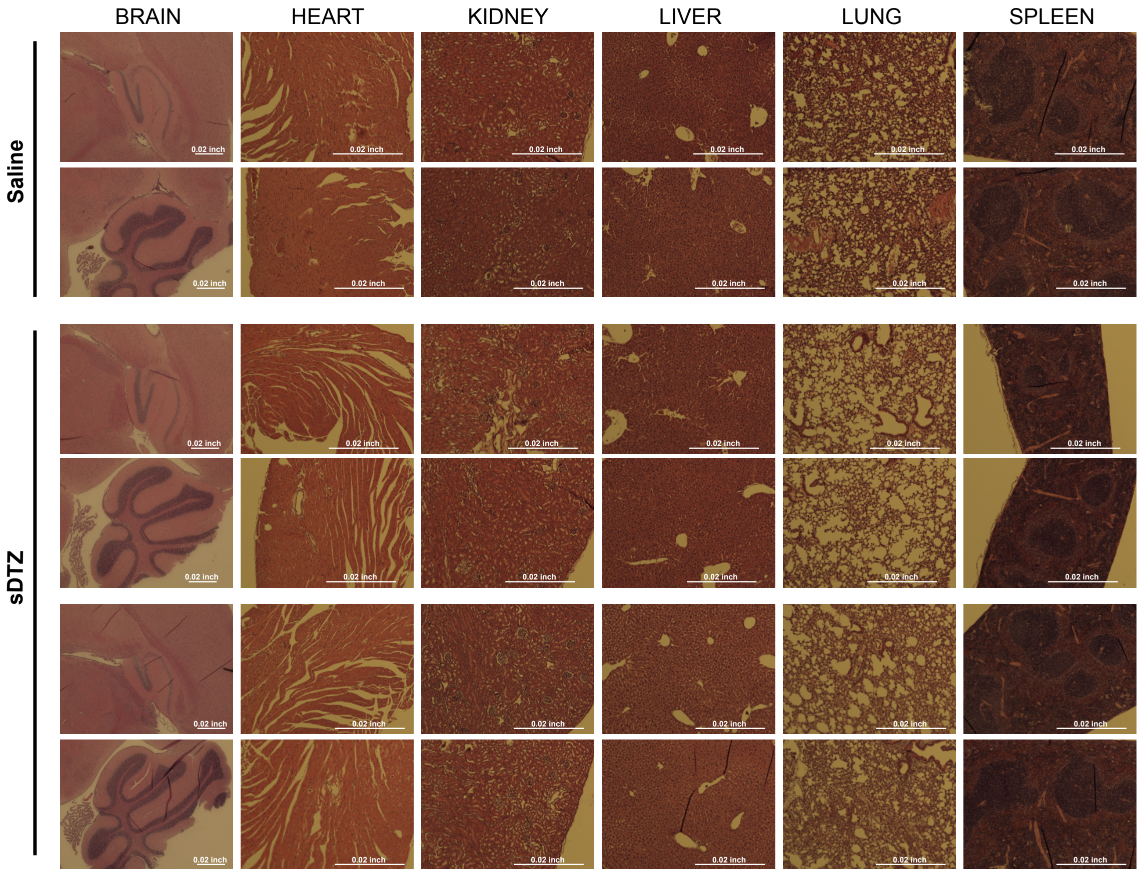

Fig. S9. Toxicity assessment of sDTZ in mice. After five consecutive days of injections with either normal saline or 25 mM sDTZ dissolved in normal saline, tissues from the brain, heart, liver, lung, kidney, and spleen of mice were harvested, sectioned, and subjected to H&E staining, suggesting no evident toxicity in both groups. For the saline group, two sections from one mouse are shown, while for the sDTZ group, a total of four sections from two mice are displayed. Scale bar, 0.02 inch.

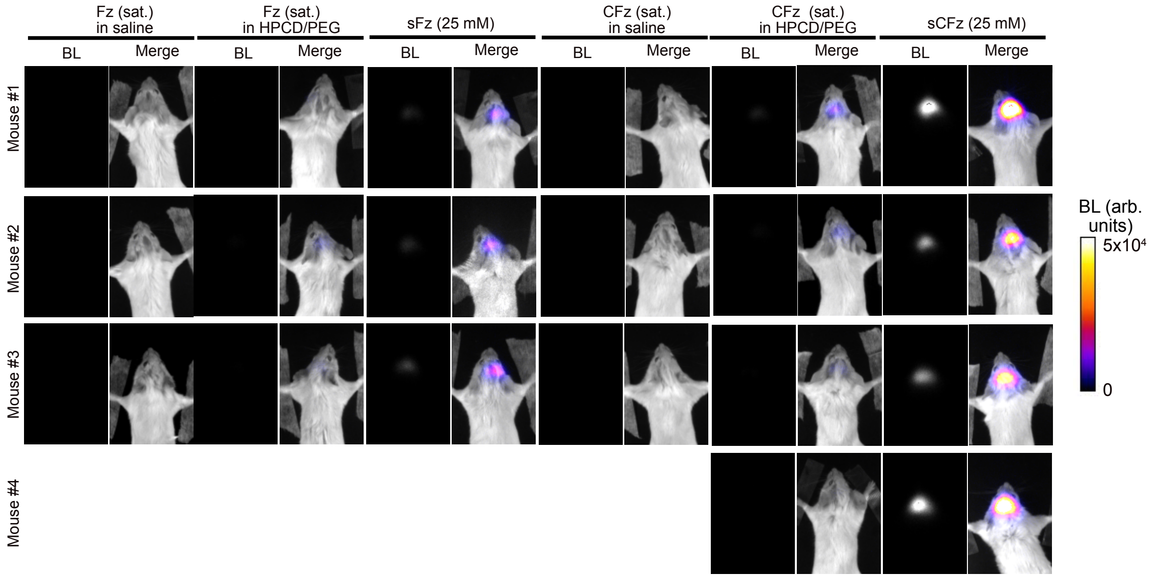

Fig. S10. Comparison of synthetic luciferins (Fz, CFz, sFz, and sCFz) for brain imaging in mice. Live mice with hippocampal Antares AAV transduction were subjected to BLI. Substrates (100 µL) at specified concentrations in the indicated injection buffers were administered via the tail vein. Normal saline was used as the delivery solvent for all sFz and sCFz experiments. Grayscale images (left) and pseudocolored images overlaid on corresponding brightfield images (right) are used to illustrate peak BL intensities from each substrate injection. A subset of these images is also displayed in Fig. 3B.

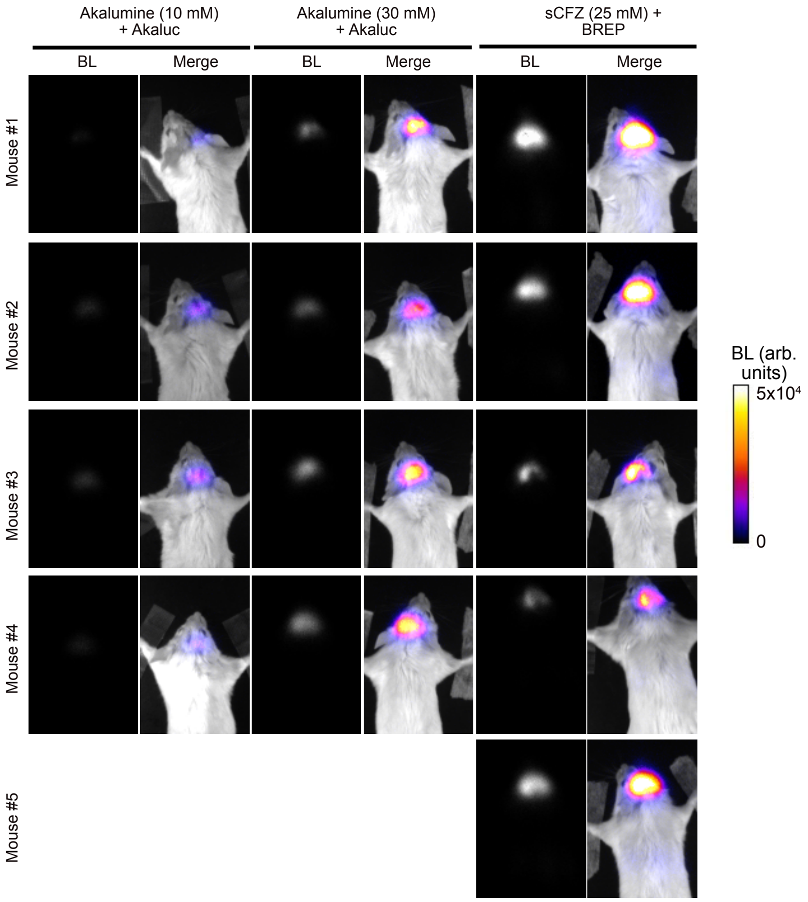

Fig. S11. Brightness test of Akaluc and BREP paired with Akalumine and sCFz, respectively. Live mice with hippocampal Akaluc or BREP AAV transduction were subjected to BLI. Substrates (100 µL) in saline at specified concentrations were administered via the tail vein. Grayscale images (left) and pseudocolored images overlaid on corresponding brightfield images (right), illustrating peak BL intensities from each substrate injection, are presented. A group of these images are also showcased in Fig. 4A.

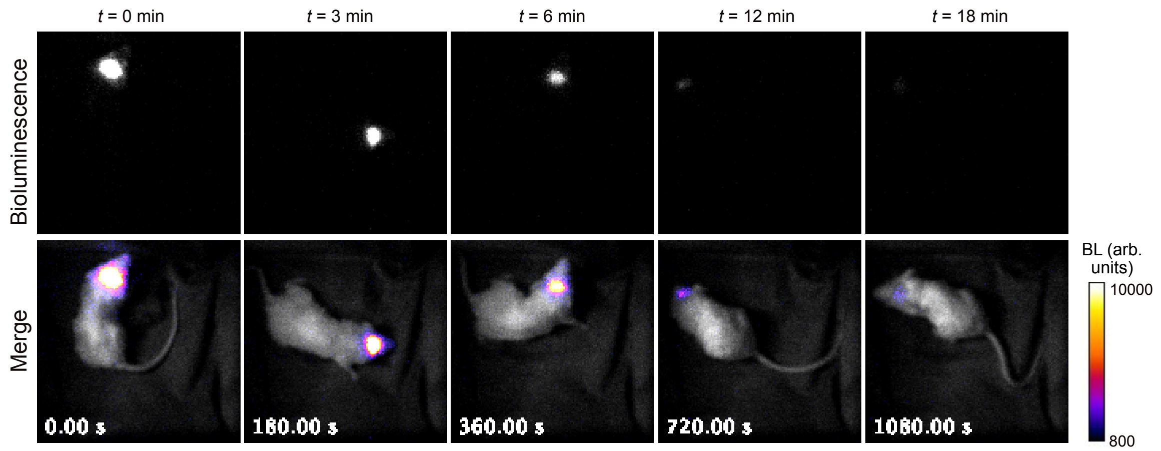

Fig. S12. High-frame video-rate imaging of a mouse with hippocampal BREP luciferase expression. The mouse was injected with sDTZ via tail vein right before imaging. Brightfield and bioluminescent images (15-ms exposure time for each) were alternately acquired using an EMCCD camera. Shown are bioluminescence (top row) and pseudocolored bioluminescence overlayed on brightfield images (bottom row) at the indicated time points. Results for the same experiments are also presented in Fig. 4D.

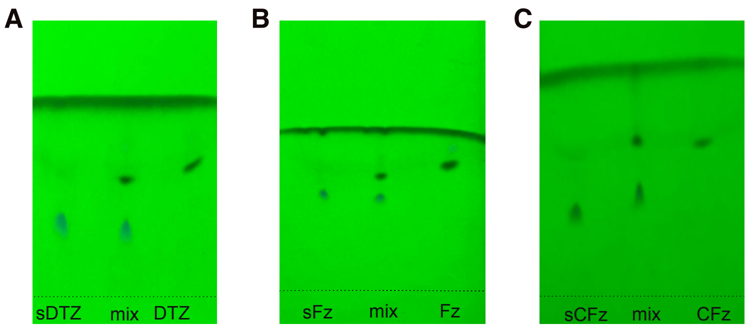

Fig. S13. TLC (DCM/methanol = 10:1) to verify the purity of sDTZ (A), sFz (B), and sCFz (C) post-purification. Migration comparison was conducted with DTZ, Fz, and CFz, respectively. The samples co-spotted in the middle lanes consisted of a mixture of PEGylated and unPEGylated luciferins.

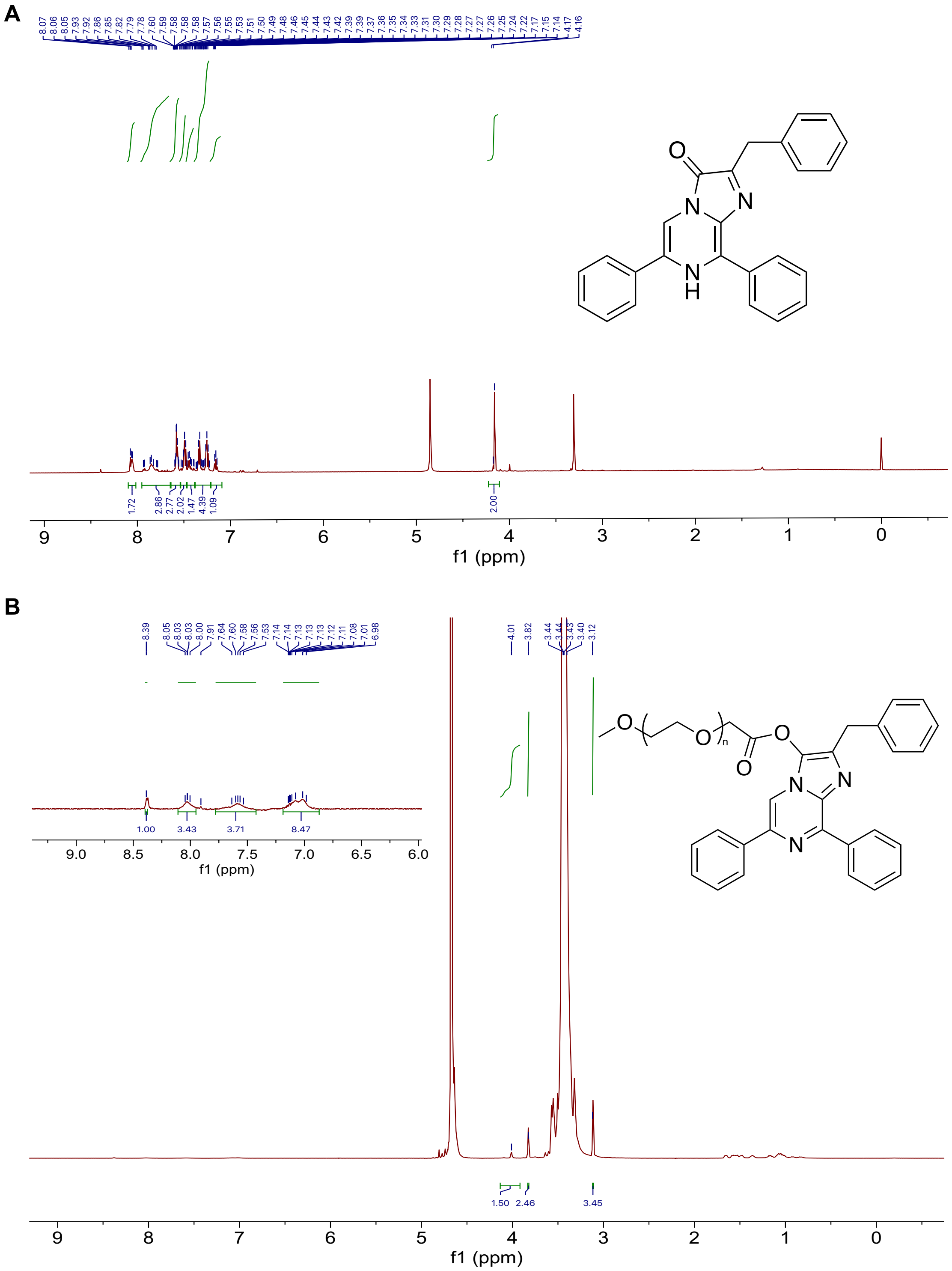

Fig. S14. ^{1}H-NMR spectra for compounds DTZ (**A**) and sDTZ (**B**).

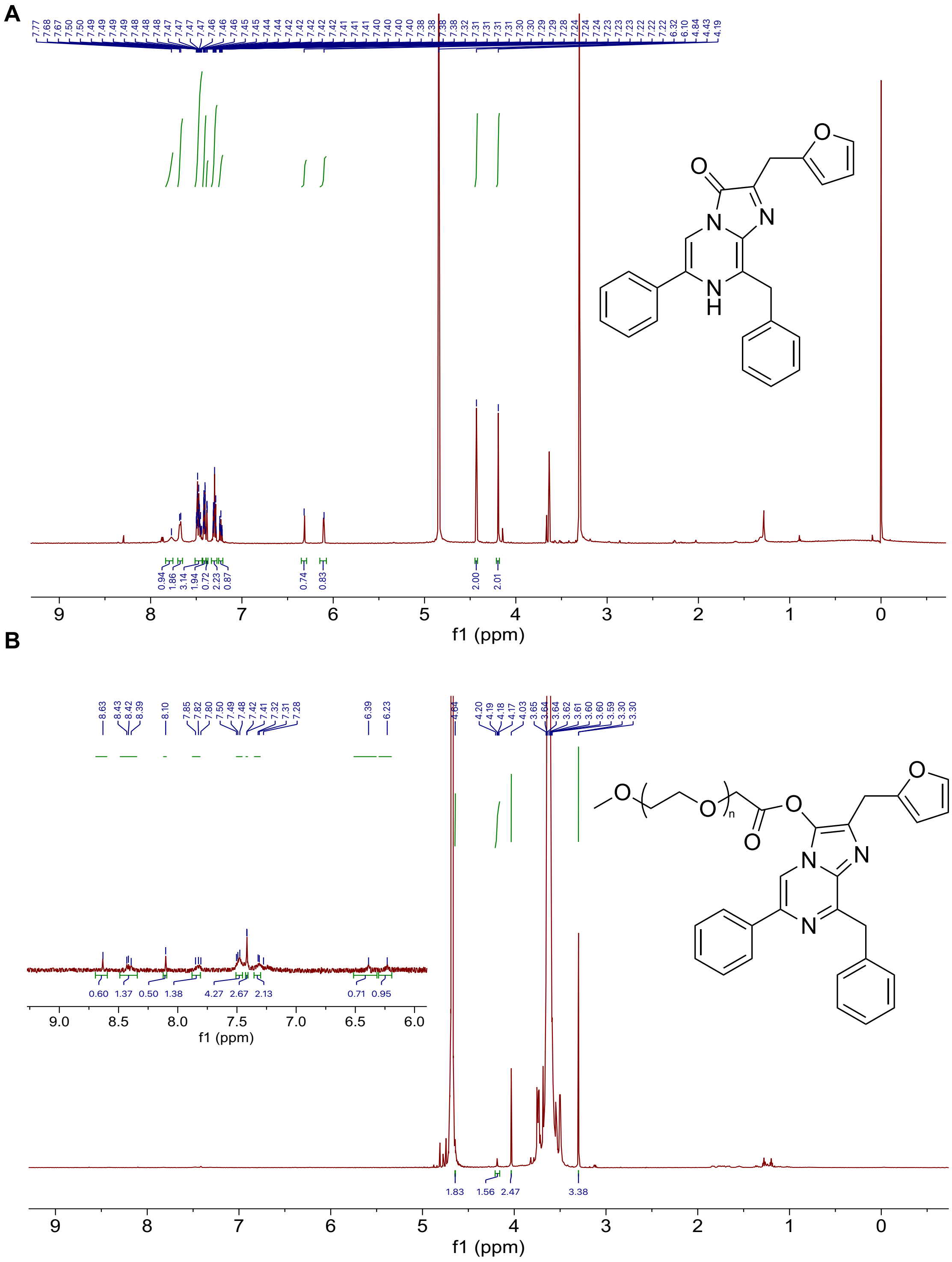

Fig. S15. ^{1}H-NMR spectra for compounds Fz (**A**) and sFz (**B**).

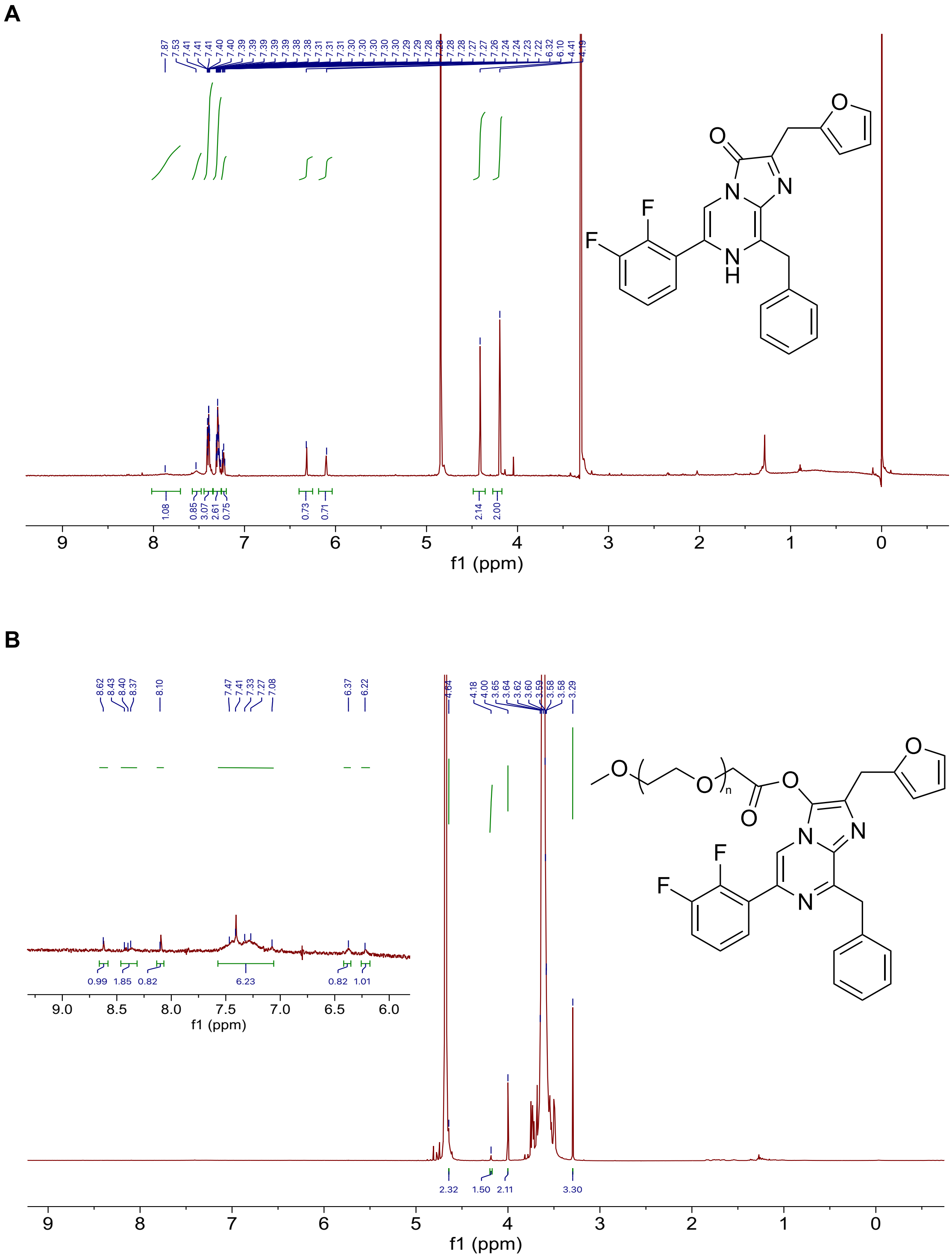

Fig. S16. ^{1}H-NMR spectra for compounds for CFz (**A**) or sCFz (**B**).

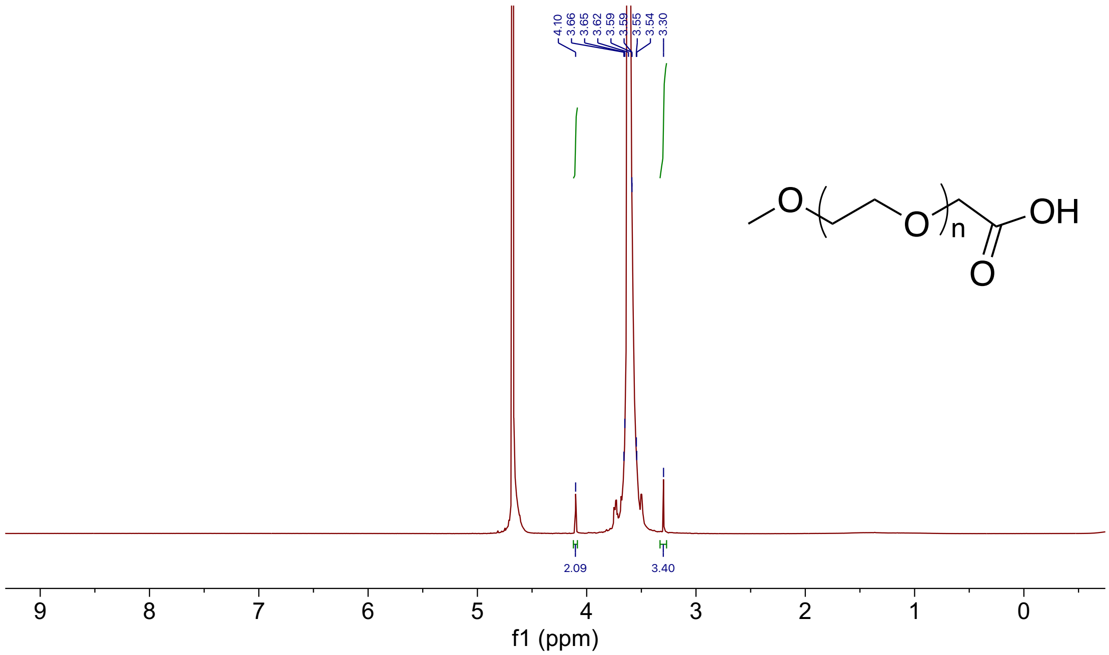

Fig. S17. ^{1}H-NMR spectra for the starting material, methoxy PEG10k acetic acid.

Table S1. Quantitative comparison of luciferase-luciferin combinations presented in Figs. 1-4 in terms of *in vivo* bioluminescence brightness and signal duration.

	**Conditions**	**Signal decay half-life (s)**	**Relative brightness at t = 0 min***	**Relative brightness at t= 15 min***	**Relative brightness integrated over 15 min***
**Brain**	BREP+DTZ (sat.) in saline	25	1	1	1
	BREP+DTZ (2.5 mM) in HPCD/PEG	30	47.4	2.4	7.3
	BREP+ETZ (6.8 mM) in HPCD/PEG	45	81.7	3.6	14.8
	BREP+sDTZ (5 mM) in saline	60	67.6	3.9	14.1
	BREP+sDTZ (10 mM) in saline	90	194.7	11.5	47.6
	BREP+sDTZ (15 mM) in saline	105	186.3	15.4	54.3
	BREP+sDTZ (25 mM) in saline	140	260	22.3	88
	Antares+Fz (sat.) in saline	25	1.1	1.2	1
	Antares+Fz (3 mM) in HPCD/PEG	120	2.7	1.3	1.3
	Antares+sFz (25 mM) in saline	135	26.3	2.9	7.5
	Antarea+CFz (sat.) in saline	25	1	1.2	1
	Antarea+CFz (3 mM) in HPCD/PEG	90	6.4	1.5	2.1
	Antares+sCFz (25 mM) in saline	170	153.8	10.9	52
	BREP+sCFz (25 mM) in saline	45	171.8	6.5	27.6
	Akaluc+AkaLumine (10 mM)	450	18.7	13.7	16.5
	Akaluc+AkaLumine (30 mM)	470	76.8	49	50.6
**Liver**	BREP+DTZ (sat.) in saline	30	1	1	1
	BREP+sDTZ (5 mM) in saline	40	275	11.7	51
	BREP+sDTZ (10 mM) in saline	40	726	27.7	139
	BREP+sDTZ (15 mM) in saline	75	728	36.8	194
	BREP+sDTZ (25 mM) in saline	240	952	141.5	482

* The brightness values were normalized to the BREP+DTZ (sat.) in saline group for both the brain and liver imaging experiments.

Legend for Movie S1

Movie S1: High frame-rate brightfield and bioluminescence imaging of a mouse with hippocampal BREP luciferase expression for over 18 min.
